# Scanning electron microscopy, morphometric and energy dispersive X-Ray analysis of cephalothoracic structures exploring defensive and sensory features in kuruma shrimp (*Marsupenaeus japonicus* Spence Bate, 1888)

**DOI:** 10.1186/s40850-024-00219-7

**Published:** 2024-11-13

**Authors:** Mohamed A. M. Alsafy, Samir A. A. El-Gendy, Hanan H. Abd-Elhafeez, Soha Soliman, Atef Erasha, Safwat Ali, Karam Roshdy, Ahmed M. Rashwan

**Affiliations:** 1 Department of Anatomy and Embryology, Faculty of Veterinary Medicine, Alexandria University, Abis 10 P.O. 21944, Alexandria, Egypt; 2 Department of Cell and Tissues, Faculty of Veterinary Medicine, Assiut University, Assiut, 71526 Egypt; 3 Department of Histology, Faculty of Veterinary Medicine, South Valley University, Qena, 83523 Egypt; 4 Department of Anatomy and Embryology, Faculty of Veterinary Medicine, University of Sadat City, Sadat City, 32897, Egypt; 5 Department of Anatomy and Embryology, Faculty of Veterinary Medicine, Minia University, Minya, 61519 Egypt; 6 Department of Histology and Cytology, Faculty of Veterinary Medicine, Alexandria University, Abis 10 P.O. 21944, Alexandria, Egypt; 7 Department of Anatomy and Embryology, Faculty of Veterinary Medicine, Damanhour University, Damanhour, 22511 Egypt; 8 Department of Life Science Frontiers, Center for iPS Cell Research and Application, Kyoto University, 53 Kawahara-ChoSakyo-Ku, Kyoto , Shogoin, 606-8507 Japan

**Keywords:** *Marsupenaeus japonicas*, SEM–EDX, Antenna, Scaphocerite, Setae

## Abstract

**Background:**

Kuruma shrimp (*Marsupenaeus japonicus*) is a commercially important crustacean and a valuable global food source. This study employed scanning electron microscopy (SEM) to explore the morphology and morphometric features of the *Marsupenaeus japonicus* cephalothoracic structures, including antennules, antennas, scaphocerite, rostrums, and eye stalks. The primary focus was on understanding the role of each part, especially through the examination of setae, which are crucial for chemoreception and defense. Additionally, energy dispersive X-ray spectroscopy (EDX) analysis was utilized to identify the elemental composition of these structures.

**Material and methods:**

The samples from the heads of fifteen *Marsupenaeus japonicus* were studied by gross morphology and morphometry, SEM, and EDX analysis. This study is the first to integrate both SEM and EDX techniques for a detailed analysis of these cephalothoracic structures, offering an innovative approach to understanding both morphological and elemental characteristics.

**Results:**

*Marsupenaeus japonicus* exhibited two antennules and two antennae. The antenna featured four basal segments: basicerite, ischiocerite, merocerite, and carpocerite, each with distinctive articulations and setae distribution. The antennule, with three segments covered by plumose setae, displayed curved cone-shaped flagellae. The scaphocerite, resembling a paddle, showcased plumose setae, while the rostrum exhibited dorsal and ventral spines, lateral grooves, and unique setal arrangements. Setal measurements across structures revealed diverse lengths and widths, indicating functional specialization. The compound eyes were connected to an optic stalk adorned with plumose setae. EDX analysis revealed higher percentages of calcium and phosphorus in the spear-like structures of the scaphocerite, rostrum, and antenna, respectively.

**Conclusion:**

This investigation provides a thorough examination of the intricate morphological features of the cephalothoracic region of *Marsupenaeus japonicus*, shedding light on its sensory and defensive capabilities. The novel application of both SEM and EDX not only deepens our insights into these structures but also lays the groundwork for future studies using this dual approach to explore crustacean morphology, with potential advantages for sustainable aquaculture and the conservation of marine ecosystems.

## Introduction

### Economic significance of Penaeid shrimp

Penaeid shrimp, recognized as one of the most economically crucial crustaceans, is a vital global food source. It plays a pivotal role in supporting the economy by providing a significant source of income for fisheries and aquaculture, as well as contributing to the provision of essential animal protein and foreign exchange for developing countries [1]. Shrimp fisheries serve as a food supply and an economic resource in Egypt and other developing countries. The commercial shrimp species belongs to the *Penaeoidea* superfamily, which has five *Penaeidean* groups. The *Penaeidae* superfamily's marine shrimp account for almost one-third of the world's commercial shrimp [2–4].

*Marsupenaeus japonicus* is of significant economic interest to the aquaculture and fisheries industries in Japan, Australia, Southeast Asia, and Mediterranean countries such as Italy, Turkey, and Egypt. The Dibah Triangle area of the Port-Said Governorate in Egypt has served as a key aquaculture location for shrimp production, including species such as *L. vannamei*, *Marsupenaeus japonicus*, and *P. semisulcatus* [5–8]. Additionally, *Marsupenaeus japonicus* plays a vital role in the fisheries economy of Japan and several Southeast Asian nations [9].

### Taxonomic and morphological characteristics of *Marsupenaeus* japonicus

*Marsupenaeus japonicus*, also known as the kuruma shrimp, kuruma prawn, or Japanese tiger prawn, is the only species found in the genus Marsupenaeus [10–12]. It is found naturally in the Indo-West Pacific's bays and seas, but as a Lessepsian migrant, it has also reached the Mediterranean Sea [13]. It is one of the largest prawn species and, thus, one of the most economically important members of the family Penaeidae, with a significant presence in aquaculture [14, 15].

An adult healthy *Marsupenaeus japonicus*, approximately 15 g in weight and measuring 10–12 cm in length, was obtained from a seafood market in Hangzhou, China [16]. The adult stage of *Marsupenaeus japonicus* is considered to commence when individuals reach a length of 10–12 cm. Additionally, for our study, we included male individuals in the adult age range of 11.7 to 13.8 cm, ensuring a comprehensive examination of the morphological features across this size spectrum. It's noteworthy that males of *Marsupenaeus japonicus* can grow to be 17–19 cm long, while females can attain lengths of 22–27 cm and weigh up to 130 g, making it one of the largest species in the Penaeidae family [13, 17]. *Marsupenaeus japonicus* displays a distinctive morphology with a pale body marked by brown bands across the back. The walking appendages, or pereiopods, are pale yellow near the bases and blue near the tips, while the swimming appendages, or pleopods, are similarly colored [13, 17].

### Defensive mechanisms in different crustaceans

The rostrum of *Marsupenaeus japonicus* is a notable feature, bearing 8–10 spines on top and one or two spines on the bottom [13, 18]. The shrimp uses its rostrum to strike at an opponent from the front or side as it swiftly approaches, and it occasionally employs its antennae to nudge the opponent multiple times. Both the rostrum and antennae are part of the shrimp's defense mechanisms [19].

In freshwater shrimp, *Macrobrachium crenulatum*, defensive responses involve the utilization of the rostrum and chelae as mechanisms against predators [20]. The elongated rostrum in *Xiphocaris elongata* serves as an effective defense against *Atya monticola* throughout various stages of predator–prey interactions, providing benefits in antipredator strategies [21]. Porcellanids exhibit highly elongated anterior and posterior carapace spines, enabling smooth lateral swimming, with studies indicating that such long spines contribute to deterring predators [22]. Moreover, the firmly extended spinous scaphocerite may function as a defense mechanism by increasing the required gape of the predator's mouth to ingest its prey. The increased surface area of the spinous scaphocerite serves a defensive purpose in predator avoidance [23].

### Functional diversity and mechanosensory roles of setae

The cuticular extensions had shafts with varying shapes and sizes known as sensilla or setae, which act as chemoreceptors or mechanoreceptors; these receptors were the primary methods used by decapod crustaceans to recognize food particles [24–26]. The positions and number of setae on decapod crustacean appendages were considered important comparative taxonomic features [27]. The setae, or tiny hairs on the legs of a shrimp, serve various functions depending on the specific appendage. They can be used for filtering food through the water and pushing it toward the mouth, as well as for grooming and detecting movement in the water. Additionally, setae play a role in propelling the shrimp through the water and can be involved in incubating fertilized eggs. Furthermore, setae are important for removing fouling, optimizing sensory reception, and aiding in movement in crustaceans [10, 28]. The diversity of setae found on different body parts of crustaceans correlates with specific functional outcomes such as feeding, grooming, and locomotion [29].

Moreover, crustacean setae have been identified as having various sensory functions, encompassing chemosensory attributes (both olfactory and gustatory), mechanosensory capabilities (involving tactile and vibration sensitivity), osmosensory responses, or a combination of these modalities. However, existing data is primarily derived from studies conducted on decapods [30, 31]. Beyond their sensory roles, most setae serve crucial mechanical functions during behaviors such as locomotion, digging, grooming, and feeding, particularly those on the appendages. The mechanical functionalities of setae appear to be closely linked to their size, shape, location, and the ultrastructure of the cuticle. Studies have revealed, for instance, that setae on appendages used for swimming tend to be predominantly feather-like (plumose setae) [26, 32].

Crustaceans' chemical sense played a role in various social communications, such as food discovery, assessment, and navigation [33]. Both the antennular flagella and the presence of aesthetasc (olfaction) and other chemosensory setae on the antennules contributed to odor activation [34]. The aesthetasc hairs of decapods are innervated by approximately 130 bipolar sensory neurons [35]. Scanning electron microscopy on cuticle outgrowths called setae (which contain sensilla) in crustaceans is beneficial for understanding how crustacean species sense their environment and for taxonomic studies [36].

Despite the economic and ecological significance of *Marsupenaeus japonicus*, detailed studies on the morphology and elemental composition of its cephalothoracic structures, particularly in relation to their functional roles, remain scarce. Previous research has primarily focused on the behavioral aspects of this species, leaving a gap in our understanding of its microstructural features, especially the setae, which play a crucial role in chemoreception and environmental interaction.

Our study aims to address this gap by using SEM and EDX to provide a comprehensive analysis of the cephalothoracic structures of *Marsupenaeus japonicus*, including the antennules, antennae, scaphocerite, rostrum, and eyestalks, highlighting their morphological intricacies and elemental composition. Our primary focus is on understanding the functional aspects of these structures, particularly in the context of defense mechanisms, with a specific emphasis on the diverse types of setae found on these components in the Kuruma shrimp. Additionally, we aim to explore how these different setae types are adapted to the shrimp's habitat and how they respond to environmental conditions. By examining the elemental composition of each part, we seek to elucidate their roles and functions in the defense mechanisms of *Marsupenaeus japonicus*.

To our knowledge, no previous study has combined both morphological and elemental analyses to provide such a detailed understanding of these critical cephalothoracic structures in *Marsupenaeus japonicus*. This integrative approach not only offers new insights into the functional morphology of this economically valuable species but also sets a new precedent for future research, emphasizing the importance of coupling microstructural analysis with elemental characterization to fully appreciate the biological and ecological roles of cephalothoracic structures in crustaceans.

## Material and methods

### Samples

Fifteen male *Marsupenaeus japonicus* shrimp were collected from the Damietta Governorate in the Mediterranean Sea. Prior to any handling or measurements, the shrimp were anaesthetized using clove oil as an anesthetic agent, following established protocols for shrimp anaesthesia [37]. The clove oil was diluted in seawater to a concentration of 100 mg/L, and the shrimp were immersed in this solution for approximately 5–10 min until full anesthesia was achieved. This ensured the shrimp were immobilized for accurate morphometric evaluations and minimized any stress or movement during subsequent steps.

Following anesthesia, the shrimp were euthanized and immediately immersed in 4% phosphate-buffered formalin for tissue fixation. The samples were preserved overnight at 4 °C to ensure adequate fixation. After fixation, the shrimp were rinsed three times with 0.1 M phosphate-buffered saline (PBS, pH 7.4) to remove excess formalin and prepare them for further analysis, preventing potential artifacts during subsequent microscopic or chemical analysis. The selection of fifteen shrimp was based on their homogeneous gender composition (all male), allowing us to standardize the morphometric evaluation across all individuals. This sample size was deemed sufficient for ensuring statistical relevance in evaluating the structural components of the cephalothoracic region.

### Gross morphology

Five heads of *Marsupenaeus japonicus* were grossly examined from each side, and photographs were captured using a quad-camera system. This system included two 2-megapixel cameras, one 8-megapixel camera, and one 12-megapixel camera, allowing us to obtain highly detailed and magnified images [38]. The camera setup used for this study was the AmScope MU Series USB 2.0 Digital Camera, manufactured by United Scope LLC**,** which is commonly employed for biological imaging [39, 40]. This system provided high-resolution images that effectively highlighted key morphological features across the cephalothoracic structures. The cameras were mounted on a microscope stand and calibrated to ensure uniform image quality across all specimens [41].

### Scanning *electron* microscopy

Cephalothoracic specimens from five *Marsupenaeus japonicus* heads were prepared for SEM analysis using a standardized fixation and preparation protocol to preserve surface morphology and ultrastructure. Initially, the specimens were fixed in a solution of 2% paraformaldehyde and 1.25% glutaraldehyde in 0.1 M sodium cacodylate buffer (pH 7.2) at 4 °C for 24 h. This dual fixation method ensured thorough preservation of tissue structures, critical for high-resolution SEM imaging [42].

Following fixation, the samples were rinsed three times with 0.1 M sodium cacodylate buffer containing 5% sucrose to maintain cellular integrity. Tannic acid was briefly applied to enhance membrane contrast, with exposure time and concentration carefully controlled to prevent interference with the subsequent energy-dispersive X-ray spectroscopy (EDX) analysis. After tannic acid treatment, the samples were rinsed with sodium cacodylate buffer to remove any remaining traces of the acid [43].

To ensure complete dehydration, the specimens were passed through a graded ethanol series (50%, 70%, 80%, 90%, 95%, and 100%), with each step lasting 15 min [44, 45]. The dehydrated specimens were then subjected to critical point drying using a Tousimis AutoSamdri-815B unit. This process replaced the ethanol with liquid CO2, which was subsequently transitioned to its gaseous state to avoid surface tension that could cause sample deformation [46].

Once dried, the specimens were mounted on aluminum stubs with colloidal carbon adhesive for stability during imaging. They were then sputter-coated with a gold–palladium layer using a Quorum Q150R S sputter coater to enhance electrical conductivity and prevent charging under the electron beam [47–49].

Imaging was performed using a JEOL JSM-IT200 scanning electron microscope, operated at 15 kV, at the electron microscopy unit of the Faculty of Science, Alexandria University, Egypt [50, 51].

### Morphometric analysis

We utilized ImageJ software (Wayne Rasband and contributors, National Institutes of Health, USA) to analyze the SEM images and measure various types of setae. Multiple measurements were taken from five male shrimp specimens to calculate the average lengths and widths of three different setae located at various sites within the cephalothoracic structures. The mean and standard error for each measurement were computed using Microsoft Excel [52–54]. We adopted a setal classification system based on established criteria outlined by [55, 56]. These sources provided comprehensive guidelines for identifying and categorizing setae in crustaceans, ensuring a standardized approach in our study.

All measurements reported in the study were conducted on intact setae. The setae were carefully examined, and only those in undamaged and clear conditions were considered for measurements [57]. This careful selection process was crucial to ensure the accuracy and reliability of the data. Additionally, multiple measurements were taken for each type of seta from five male shrimp specimens to provide robust data representation. The averages for the lengths and widths were then calculated along with the mean standard error to reflect the variability within our sample.

### SEM–EDX measurements

Energy-dispersive X-ray spectroscopy (EDX) was employed to analyze the elemental composition of the external surface of the cuticle in various cephalothoracic structures of *Marsupenaeus japonicus*. Both soft structures, such as the antenna, and hard structures, including the rostrum and the spear-like scaphocerite, were subjected to this analysis. A total of five samples were analyzed to ensure consistency and reliability of the data.

For the analysis, a JEOL JSM-IT200 scanning electron microscope equipped with an EDX detector was used, operating at an accelerating voltage of 20 kV. The sample-to-detector distance was set to 10 mm, and the data acquisition was optimized with a real-time duration of 30.97 s and a dead time of 3.00%. This setup allowed for accurate detection and quantification of the elemental composition. The analysis employed the ZAF (atomic number, absorption, and fluorescence) quantification method to correct for matrix effects and ensure precise measurements of the element concentrations.

All SEM–EDX measurements were conducted at the Electron Microscope Unit, Faculty of Science, Alexandria University, Egypt, where the equipment is regularly calibrated to ensure optimal performance [57, 58]. The integration of this technique provided a detailed elemental analysis of the cuticle structures, complementing the morphological data obtained through SEM imaging.

### Coloring of SEM images

We used the Photo Filter 7.2.1 program to color the SEM images to enhance the visibility of the morphological details [59, 60]. The primary goal was to improve visual contrast, making it easier to distinguish specific features and structures within the images. This enhancement facilitates better interpretation and analysis of the morphological characteristics being studied.

## Results

**Fig. 1:**
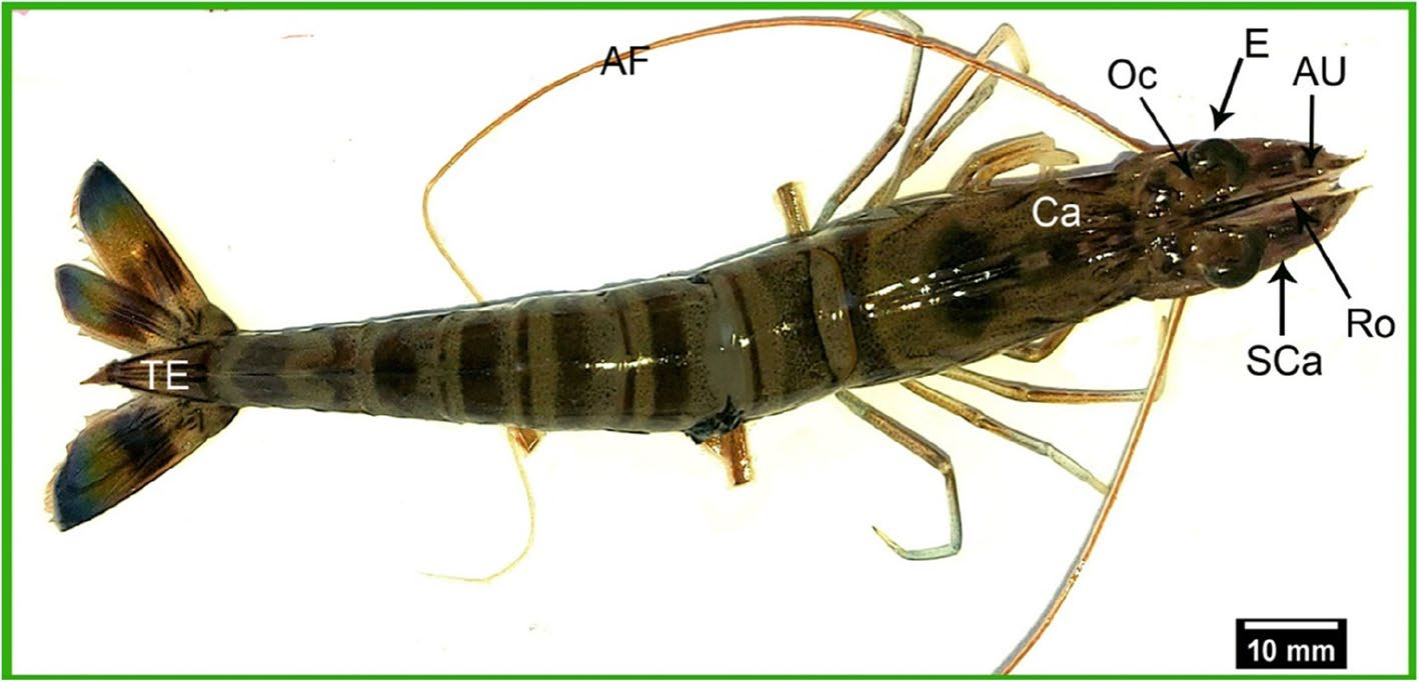
A gross dorsal view of *Marsupenaeus japonicus*, highlighting the structures of the cephalic region. The figure shows that the eyes are black and attached to the bony palate (E). It also labels the optic calathus (Oc), median rostrum (Ro), antennule (AU), antenna flagellum (AF), scaphocerite (SCa), carapace (Ca), and telsonian rostrum (TE)

*Marsupenaeus japonicus* exhibited a standard length, measured from the base of the eye to the base of the shrimp, ranged from 9 to 11 cm (9.794 ± 0.07). The total length, measured from the tip of the rostrum to the end of the telson, was 11.7 to 13.8 cm (12.5 ± 0.12). Our study focused specifically on the structures in the cephalothoracic region for a detailed investigation of SEM structures related to chemoreception and defense mechanisms, including antennules, antennas, scaphocerite, rostrums, and eye stalks (Fig. 1).

### Antennule

**Fig. 2:**
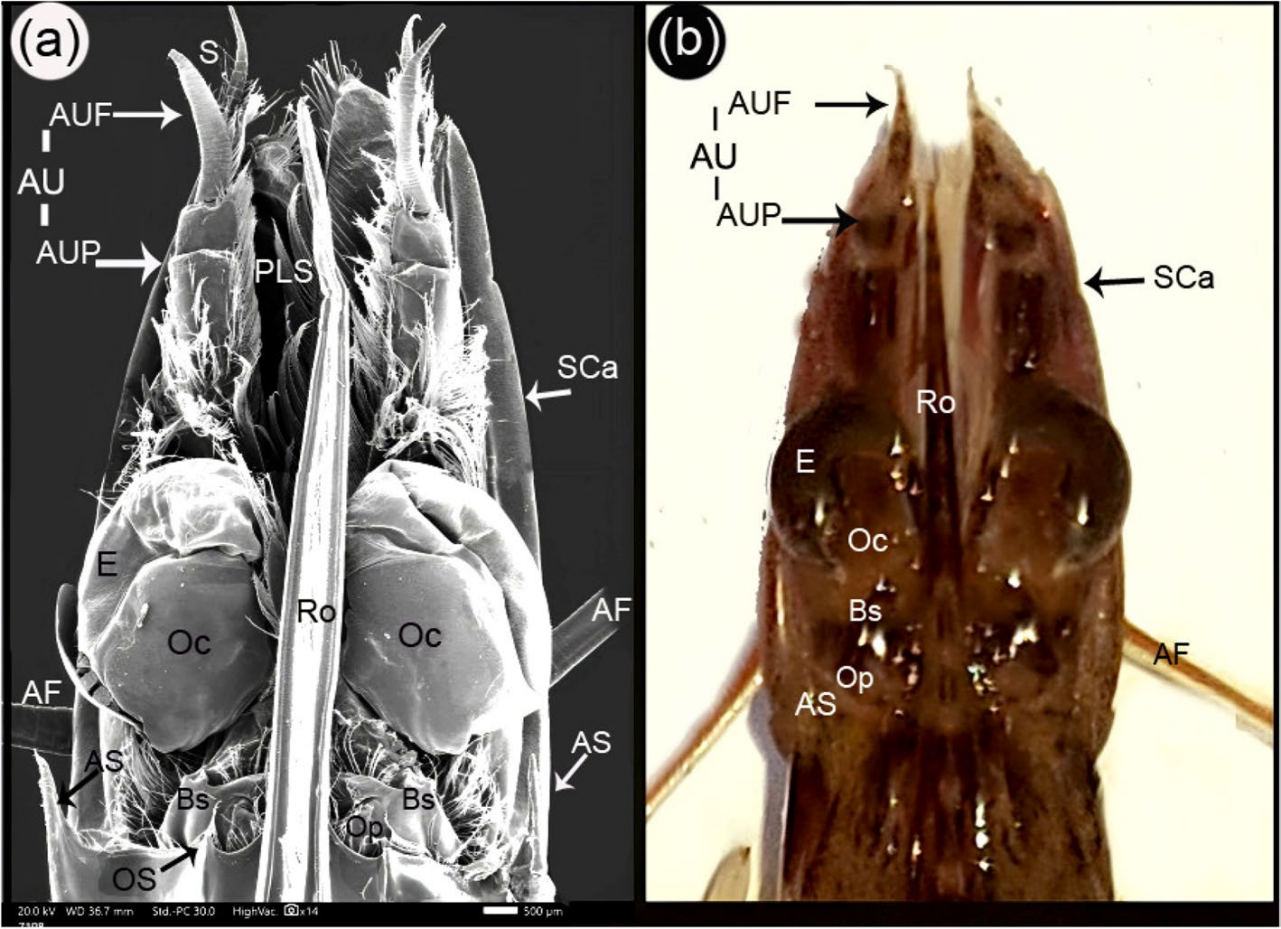
SEM dorsal view (**a**) and gross dorsal view (**b**) of the cephalic eye region of *Marsupenaeus japonicus.* The figure shows the eye attached to the bony palate (E), the optic calathus (Oc), the basal segment (Bs), the ocular plate (Op), the median rostrum (Ro), and the antennule (AU), which consists of the antennule peduncle (AUP) and the antennule flagellum (AUF). Additionally, the figure labels the setae (S), antennal flagellum (AF), scaphocerite (SCa), plumose seta (PLS), orbital spine (OS), and antennal spine (AS)

**Fig. 3:**
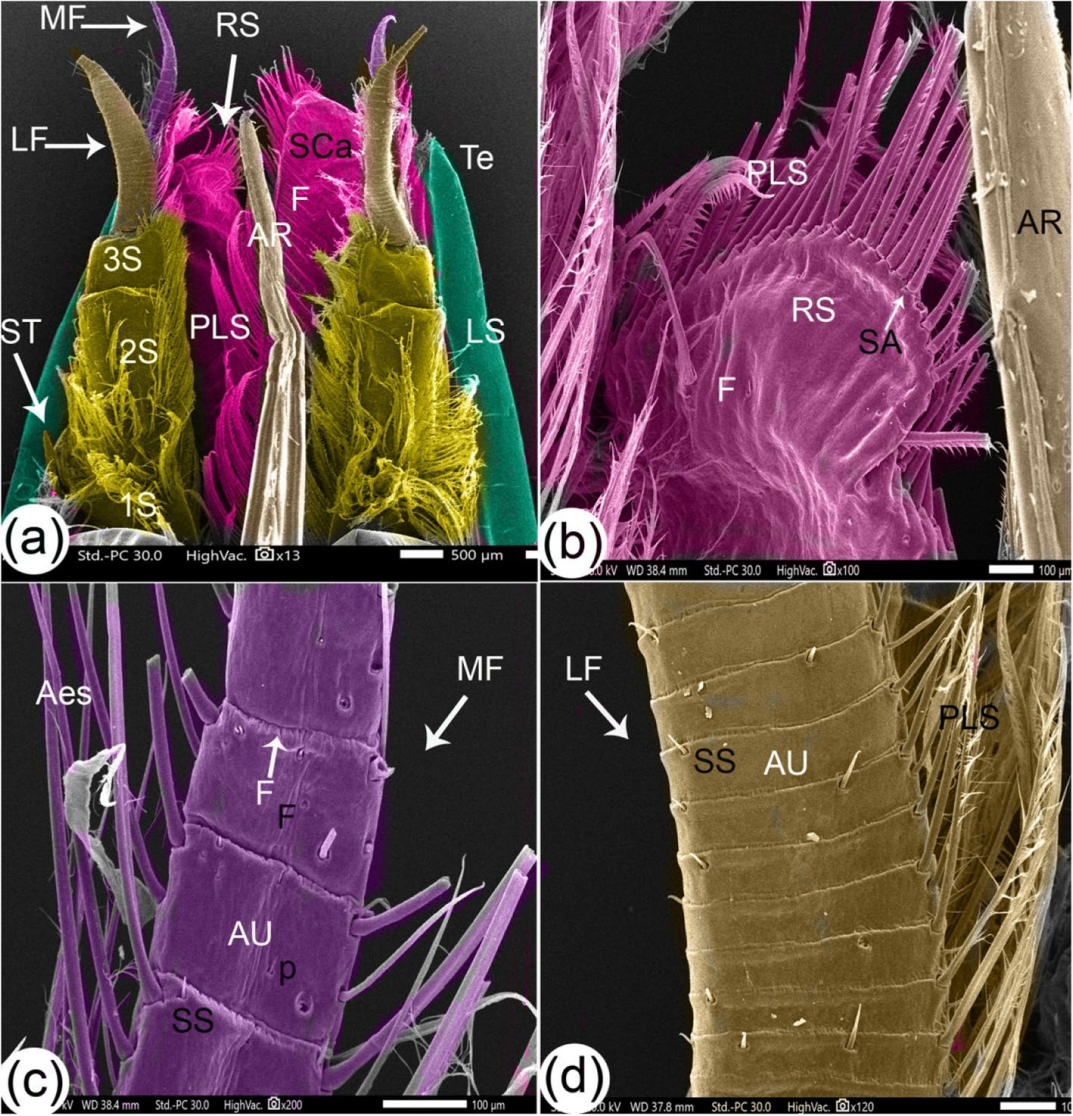
SEM images of the antennule and scaphocerite with setae structures of *Marsupenaeus japonicus*: dorsal view of the eye stalk (**a**), rostral part of the scaphocerite (**b**), medial flagellum of the antennule (**c**), and lateral flagellum of the antennule (**d**). The antennule peduncle consists of a distal segment (DS), intermediate segment (MS), and proximal segment (PrS). The distal segment is extended by stylocerite (ST) and gives rise to two flagella: the thin medial flagellum (MF) and the thick lateral flagellum (LF). The scaphocerite (SCa) is primarily oval with a spear-like lateral edge (LS) and a tapered end (Te). Other labeled features include the rostral semicircular part (RS), folds (F), long plumose setae (PLS), setae articulation (SA), apical part of rostrum (AR), annulus (AU), simple setae (SS), pores (P), and aesthetasc (Aes)

The antennule peduncles and two flagella were observed dorsal to the scaphocerite (Figs. 1 and 2b). The antennule peduncles had three segments: the distal segment being the longest, the intermediate segment being the second longest, and the proximal segment being the shortest. Plumose setae covered all three segments, and the distal segment also had a lateral stylocerite extension. The flagella were curved and cone-shaped (Figs. 2a, b, and 3a). The thin and long medial flagellum measured 3240.4 ± 63.2 µm, while the thick lateral flagellum measured 2895 ± 119.1 µm. The medial flagellum had the highest annulus width of 130.6 ± 5.4 µm. Folds appeared in the middle of the annulus and sites of articulation (Fig. 3b). Aesthetasc setae appeared on both sides of the medial flagellum (Fig. 3c). Aesthetasc setae are long, thin-walled, tubular, and smooth, with a rounded tip. They are exclusively associated with the antennule. The plumose setae exhibit simple setae on their lateral and medial sides and near the annulus articulation (Fig. 3d). Long, thin-walled shafts with setules of variable length characterized plumose setae. These setae are flexible and arranged in two or more opposing rows. In contrast, simple setae have smooth setal shafts without setules or scales, tapering to a sharp point either with or without a hole (Fig. 3d).

### Antenna

The antenna presented as a lengthy flagellum tapering towards the caudal end (Fig. 1). It measured 10.032 ± 0.10 cm in length, with a width ranging from 0.44 mm at the base to 0.08 mm at the tapered tip of the flagellum. The mean width of the flagellum at its lower third was 0.3692 ± 0.024 cm; in the middle third, it measured 0.336 ± 0.0116 cm, and in the distal third, it reduced further to 0.146 ± 0.022 cm (Fig. 1).

**Fig. 4:**
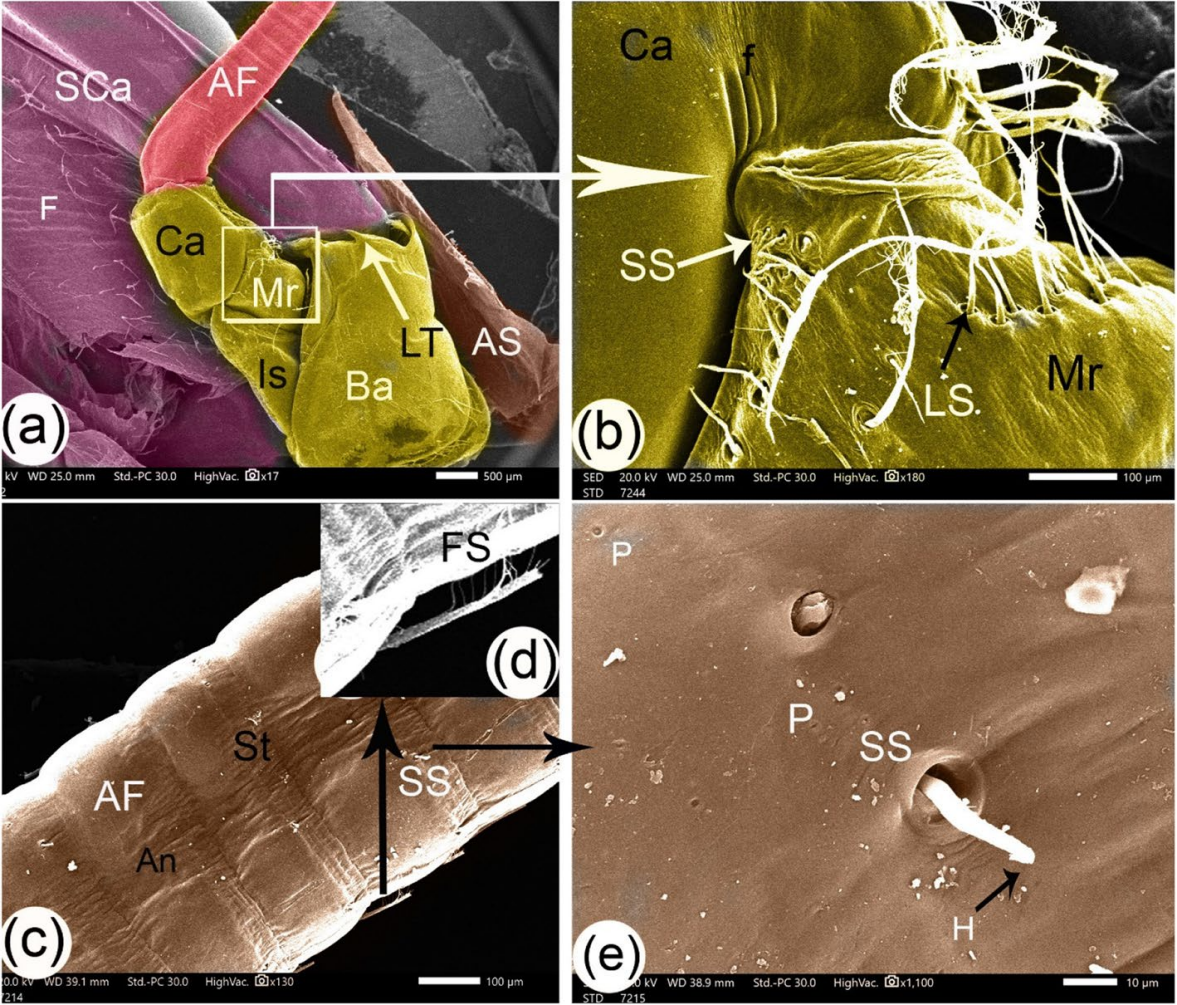
SEM images of the antenna of *Marsupenaeus japonicus*: (**a**) the full view of the antenna, (**b**) an enlargement of the merocerite from view (**a**), (**c**) the origin of the flagellum, and (**d**, **e**) enlargements from view (**c**). The antenna consists of four segments: basicerite (Ba), ischiocerite (Is), merocerite (Mr), and carpocerite (Ca). The antenna flagellum (AF) is composed of band-like straps (St) and successive bands (annulus) (An). Other labeled structures include the scaphocerite (SCa), lateral teeth (LT), three folds (**f**), small simple setae (SS), long filamentous setae (LS), a small seta with a hole at its proximal end (H), pits (P), antennal spine (AS), and unipennate feather setae (FS)

The antenna featured four strong basal segments. Basicerite, ischiocerite, merocerite, and carpocerite were the four segments; the basicerite was the largest, with a rectangular shape and lateral teeth at the upper margin (Fig. 4a). The basicerite articulated with the rectangular-shaped ischiocerite and the cubic-shaped merocerite with three folds. The latter two were articulated with the cylindrical carpocerite, which was articulated with the antenna flagellum. The setae were found on the four segments, mainly in the dorsal part of the merocerite. They consisted of small, simple setae and long filamentous setae (Fig. 4b). Filamentous setae are characterized by their slender, thread-like structures. These setae typically feature long, thin, and flexible shafts resembling filaments and may exhibit diverse arrangements (Fig. 4b). The flagellum appeared as successive bands (annulus) that connected at a constricted, band-like strap (49.04 µm in width) that was slightly folded and contained at the central apart that had a small, simple seta with a hole at its proximal end (Fig. 4c). Unipennita setae were found in the small seta that originated at the setal pocket, as well as small pits on the lateral side of the strap area (Fig. 4d and e). Unipennate setae are characterized by their feather-like structure, resembling a single-sided comb. These setae have a central shaft with thin, hair-like branches extending from one side of the shaft, creating a unidirectional arrangement similar to the teeth of a comb (Fig. 4d).

### Scaphocerite

**Fig. 5:**
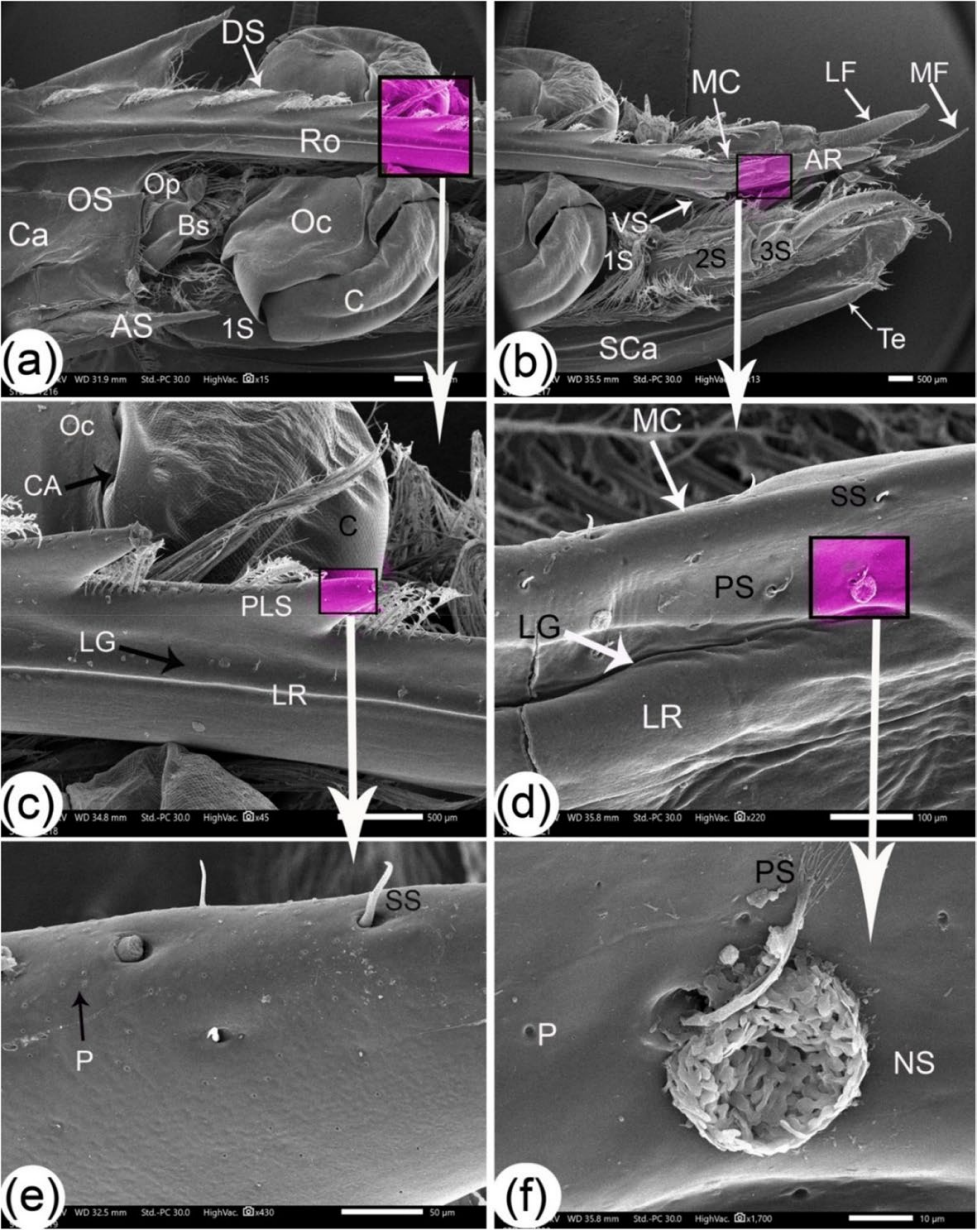
SEM images of the lateral side of the cephalic region of *Marsupenaeus japonicus*: (**a**) the caudal part of the cephalic region, (**b**) the rostral part of the cephalic region, and (**c**, **d**, **e**, **f**) enlarged views of the highlighted areas in the previous images, showing the following structures: the cornea (C), the attached bony palate, the optic calathus (Oc) with wavy connections (CA), the basal segment (Bs), and the ocular plate (Op). The carapace (Ca) features two spines: the antennal spine (AS) and the orbital spine (OS). The antennule peduncle consists of a distal segment (DS), intermediate segment (MS), and proximal segment (PrS), extending into a thin medial flagellum (MF) and a thick lateral flagellum (LF). Other labeled structures include the scaphocerite (SCa), tapered end (Te), rostrum (Ro), apical part of the rostrum (AR), dorsal spines (DS), lateral groove (LG), median crest (MC), lateral ridges (LR), ventral spine (VS), mesial crest (MC), small simple setae (SS), short plumose setae (PLS), pores (P), pappose setae (PS), and nest-like structures (NS)

The scaphocerite was a paddle-like structure ventral to the antennule and rostrum (Figs. 1, 2b). It had two parts: a long oval with a lateral edge and a spear-like shape with a tapered end; its rostral part was semicircular in shape; and oblique folds appeared on the dorsal surface near its medial side, which was more prominent than the folds on the ventral surface. In addition, long plumose setae appeared on the medial margins, and the margin of the rostral semicircular part had a pocket extension that articulated with plumose setae that act as paddles in addition to wavy folds at the dorsal surface (Figs. 2a, 3a, 5a, and b).

### Rostrum

It is a spear-like rostral carapace extension that passes between the two eyes and reaches the level of the antennule flagellum (Figs. 1 and 2b). The serrated extension from the carapace's midline extended the rostral until the rostral part of the scaphocerite had a tapered end with no dorsal spines. The rostrum's dorsal midline had 9–10 dorsal spines and one ventral spine before its pointed end and at the level of the middle of the second segment of the antennule peduncle (Fig. 5a and b). Two lateral grooves and then lateral ridges flanked the dorsal spines (Fig. 5c). The spines were obliquely directed rostrally with angles ranging from 35 to 45 degrees, with a short plumose seta on the dorsal border of each dorsal spine and simple setae and pores at the apex of the dorsal spines and lateral groove (Fig. 5d and e). While there were no setae on the ventral rostral spine, the tapered rostrum had a median crest flanked by narrow lateral grooves and then lateral ridges (Fig. 5d). The median crest's dorsal surface had simple setae and pores. At the same time, the lateral surface had pappose setae, pores, and a nest-like structure, with the margin of the socket of pappose setae at the same level as its surroundings (Fig. 5d and f). Pappose setae are characterized by short, thin-walled shafts presented at a socket. The setules, usually long and varying from thin to stout, are irregularly arranged around the shaft. These setae typically exhibit a structure where the shaft arises from a socket, and the setules extend in a manner that contributes to the overall appearance of the seta (Fig. 5d and f).

### The eyes

**Fig. 6:**
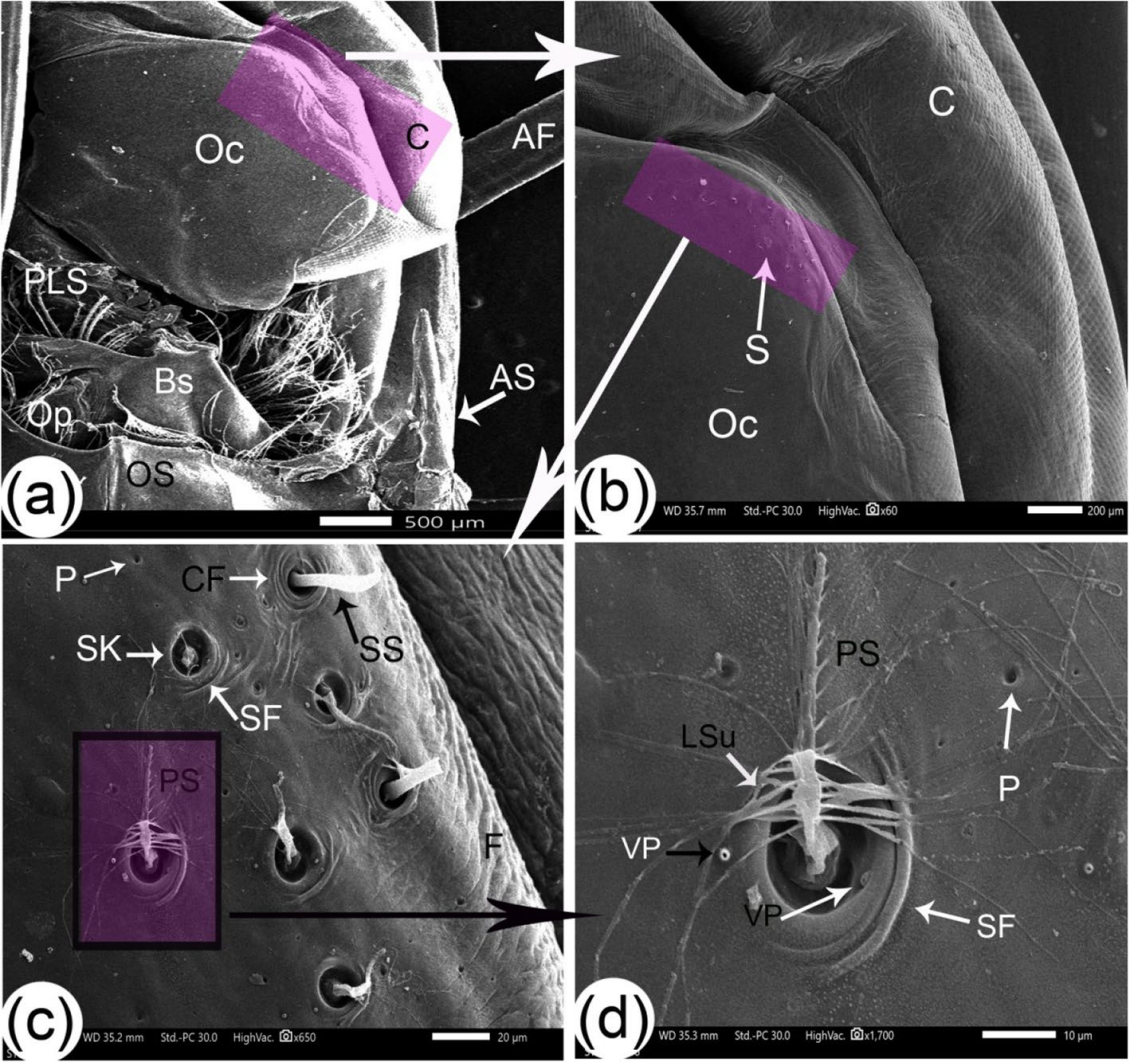
SEM images of the optic stalk with setae structures of *Marsupenaeus japonicus*: (**a**) dorsal view of the eye stalk, and (**b**,** c**, **d**) enlarged views of the highlighted areas from the previous image. The following structures are labeled: cornea (C), attached bony palate, optic calathus (Oc), basal segment (Bs), ocular plate (Op), antennal flagellum (AF), antennal spine (AS), orbital spine (OS), and various setae (S), including long plumose setae (PLS), simple setae (SS), pappose setae (PS), and long setules (LSu). Additional features include setae sockets (SK), semicircular folds (SF), complete circular folds (CF), folds (F), pits at the same level (P), and volcano-like pits (VP)

Two compound eyes appeared black-colored and hemispherical on each side of the rostrum. The eye was attached to an optic stalk that consisted of three parts: the optic calathus, basal segment, and ocular plate (Fig. 2b). The optic calathus that is attached to the eye acts as an irregular circular plate; its basal segment was L-shaped and was the largest segment, and the ocular plate was small as an extension from the carapace. Plumose setae appeared on the eye stalks (Figs. 2a and 6a). On the dorsal part of the parietal surface of the optic calathus near the cornea, there were two types of setae: simple setae and pappose setae with long setules that were longer than the shaft of the seta (Fig. 6b, c, and d). Some setae sockets were surrounded by semicircular folds toward the cornea. In contrast, other sockets had complete circular folds on the surface of this area as folds, two types of pits at the same level of the surface with different diameters, and volcano-like pits that were also present at setae sockets (Fig. 6c and d). The cornea appeared as a honeycomb shape in small units (Fig. 5c). The short orbital spine and the long wedge-shape antennule spine were extensions of the carapace behind the eye stalk (Figs. 4a, 5a, and 6a).

### Setae measurements (Table 1, Figs. 7, 8 and 9)

**Fig. 7:**
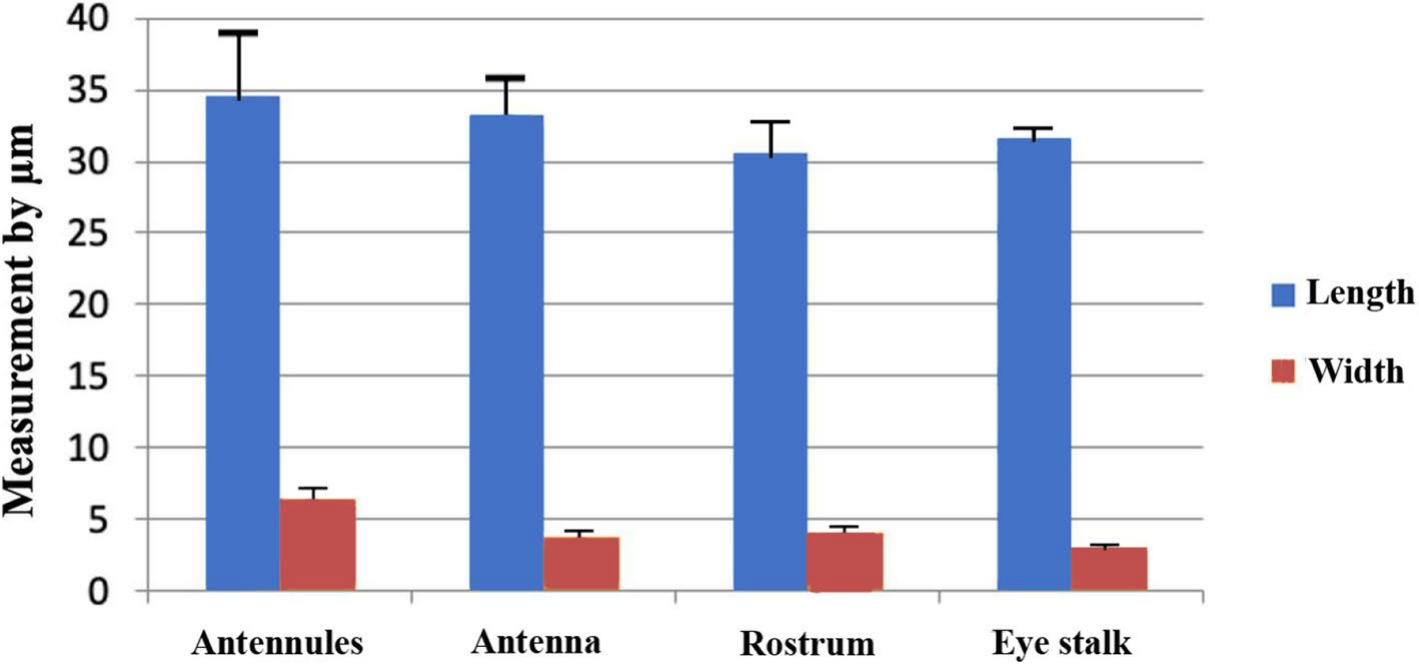
A graph illustrating the length and width of the base of simple setae at various locations on the head of *Marsupenaeus japonicus*

**Fig. 8:**
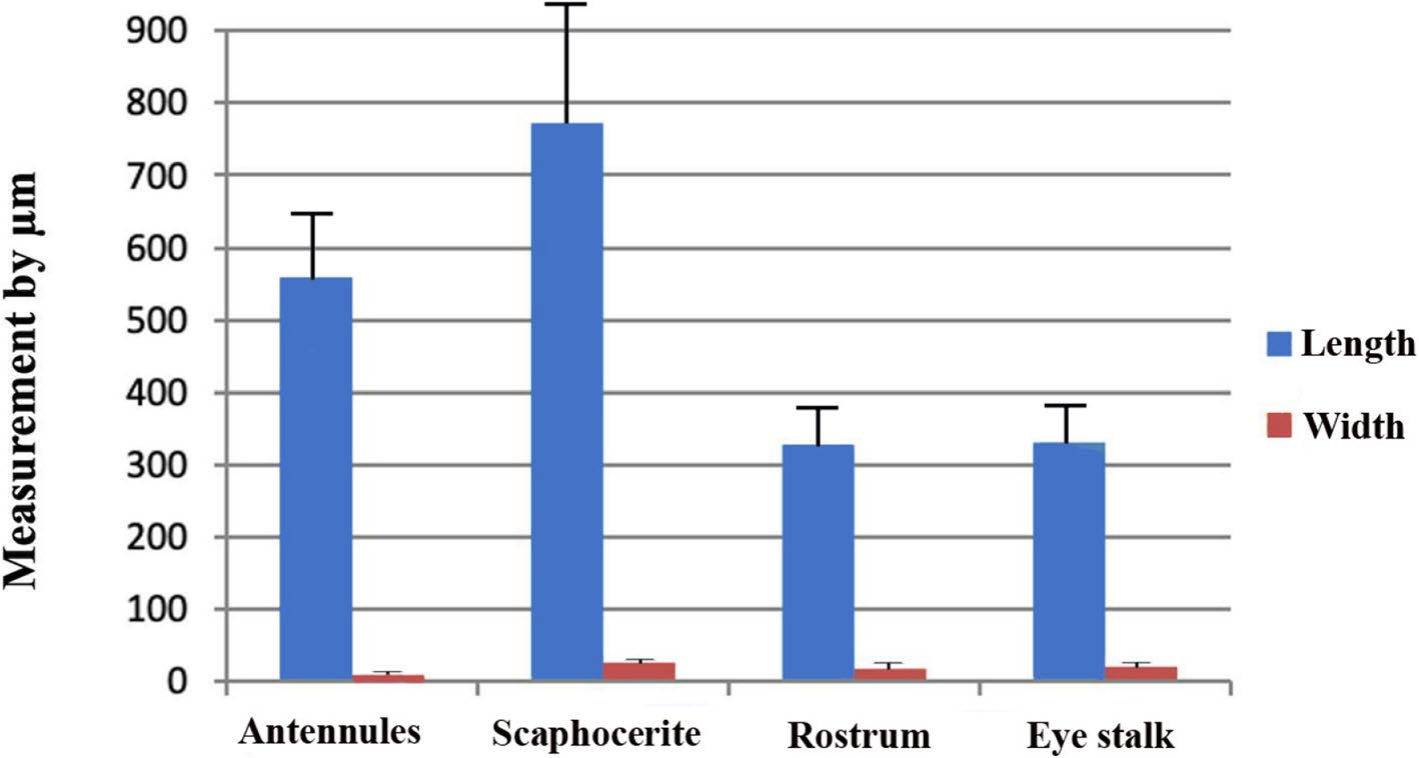
A graph illustrating the length and width of the base of the plumose setae at various locations on the head of *Marsupenaeus japonicus*

**Fig. 9:**
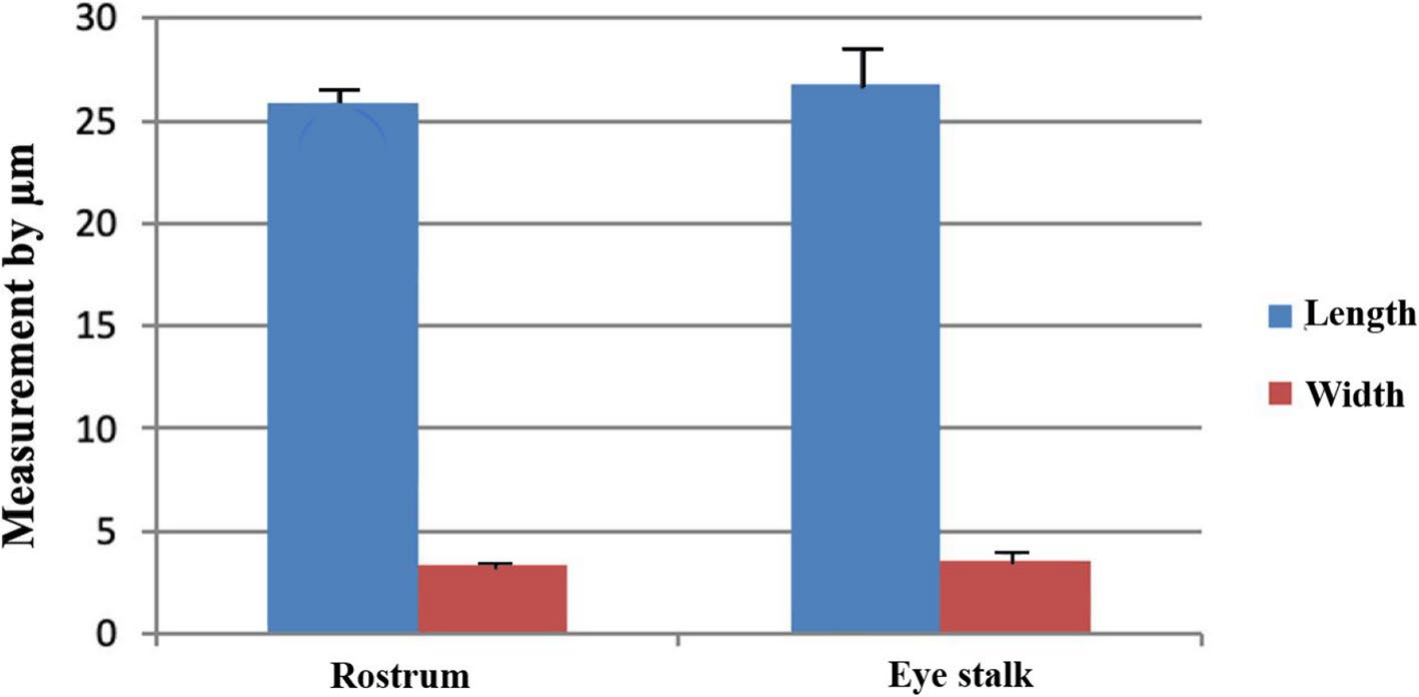
A graph illustrating the length and width of the base of the pappose setae at the rostrum and eye stalks on the head of *Marsupenaeus japonicus*

Recognizing the variations in length and width within the same seta type across different organs, the measurements of simple setae demonstrated diverse dimensions throughout the examined structures. The mean length value ranged from 34.6 ± 11.1 µm at the antennules to 30.66 ± 4.6 µm at the dorsal part of the rostrum spines. Simultaneously, the base width varied from 6.4 ± 1.3 µm at the antennules to 3.04 ± 0.2 µm at the eye stalk, displaying a caudal decrease in measurements. Plumose setae exhibited significant variations in both length and width among different structures. The mean length value ranged from 771.2 ± 236.2 µm at the scaphocerite to 328.6 ± 39.4 µm at the rostrum, with the base width showing variability from 26.2 ± 0.1 µm at the scaphocerite to 9.96 ± 1.4 µm overall. Notably, the length of plumose setae demonstrated a general decrease from ventral to dorsal structures. Pappose setae, identified at the rostrum and eye stalk at the optic calathus, displayed variations in length, ranging from 25.854 ± 1.2 µm to 26.8 ± 2.1 µm. Aesthetasc setae, exclusive to the medial flagellum of the antennule, maintained a consistent length of 390.8 ± 20.2 µm. The ratio of length to width for simple setae varied across different structures in the cephalic region, ranging from 5.41 ± 2.05 at the antennules to 10.39 ± 0.69 at the eye stalk. For pappose setae, the length-to-width ratio was nearly the same at the rostrum and eye stalk, approximately 7.6. The ratio of length to width for plumose setae varied, with the highest ratio observed at the antennules (56.17 ± 12.59), followed by the scaphocerite (29.44 ± 9.02), the rostrum (18.84 ± 2.91), and the lowest ratio at the eye stalk (16.96 ± 3.46). These variations underscore the dynamic nature of setal dimensions within distinct anatomical locations, contributing to the overall complexity of *Marsupenaeus japonicus's* morphology.

**Table 1:** Shows the length, width, and length-to-width ratios (± standard error) for different setae types at various structures in the cephalic region of *Marsupenaeus japonicus*, measured in µm

	** Types Setae **	**Length **	**Width at base **	**Length to width ratio**
Antennules	Aesthetasc	390.8 ±20.2	20.8 ±1.02	18.79 ± 1.34
	Simple setae	34.6 ±11.1	6.4±1.3	5.41 ± 2.05
	plumose setae	559.48± 97.7	9.96±1.4	56.17 ± 12.59
Antenna	Unipennita setae	93.9± 0.2	6.6 ±0.461	14.23 ± 0.99
	Filamentous	260.7±16.149	7.9 ±0.47	33.00 ± 2.83
	Small simple setae	33.3 ±7.3	3.78 ±0.27	8.81 ± 2.03
Rostrum	Plumose setae	328.6 ±39.4	17.44 ±1.7	18.84 ± 2.91
	Simple setae	30.66 ±4.6	4.02 ±0.41	7.63 ± 1.38
	Pappose setae	25.854 ±1.2	3.36±0.09	7.69 ± 0.41
Eye stalk	Simple seta	31.6 ± 0.2	3.04 ± 0.2	10.39 ± 0.69
	Pappose setae	26.8 ±2.1	3.5 ±0.26	7.66 ± 0.83
	Plumose setae	330.42 ±46.13	19.48 ±2.89	16.96 ± 3.46
Scaphocerite	Plumose setae	771.2 ± 236.2	26.2± 0.1	29.44 ± 9.02

### EDX analysis

**Fig. 10:**
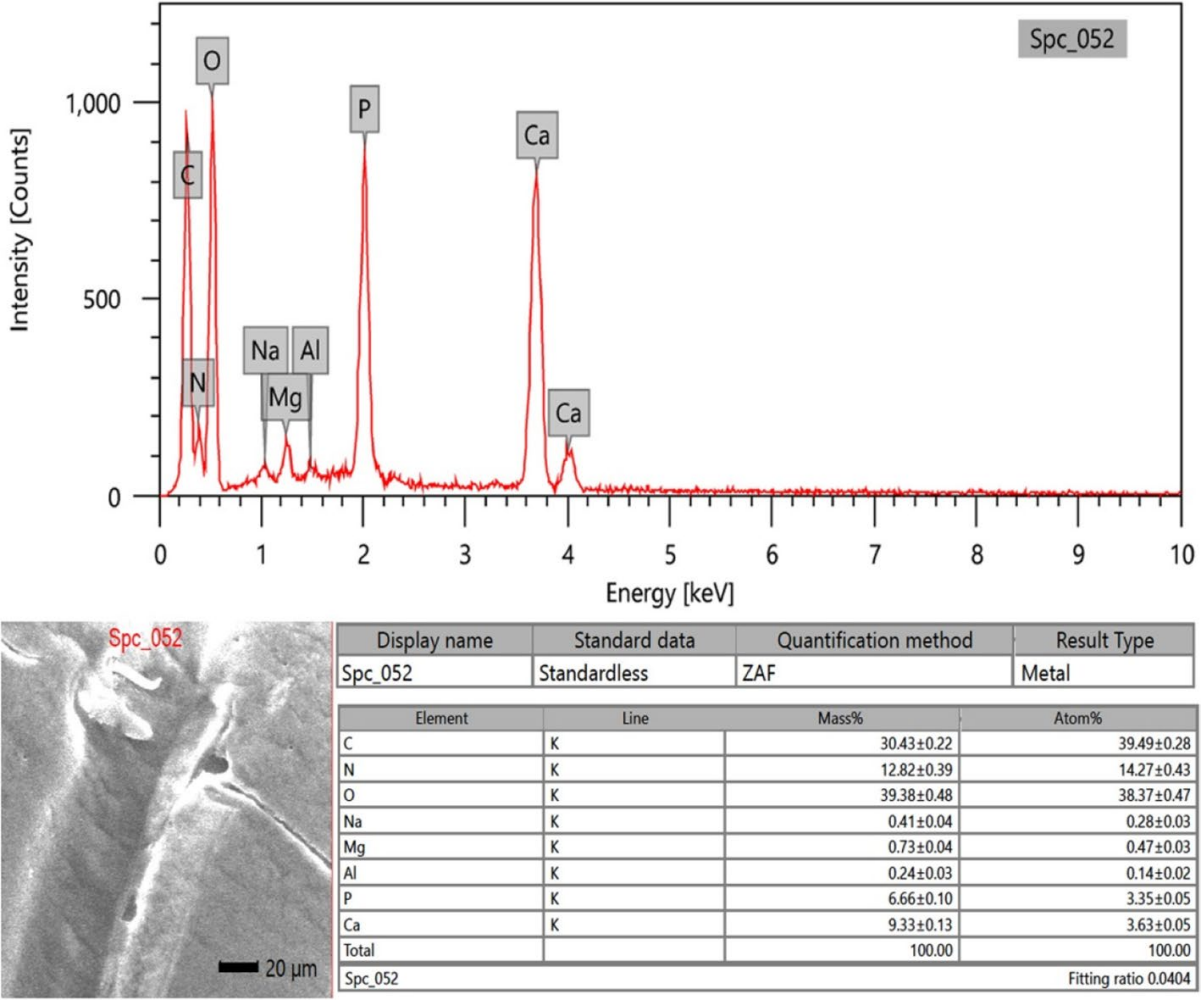
SEM–EDX image showing the elemental composition of the external surface of the antenna cuticle in *Marsupenaeus japonicus*

**Fig. 11:**
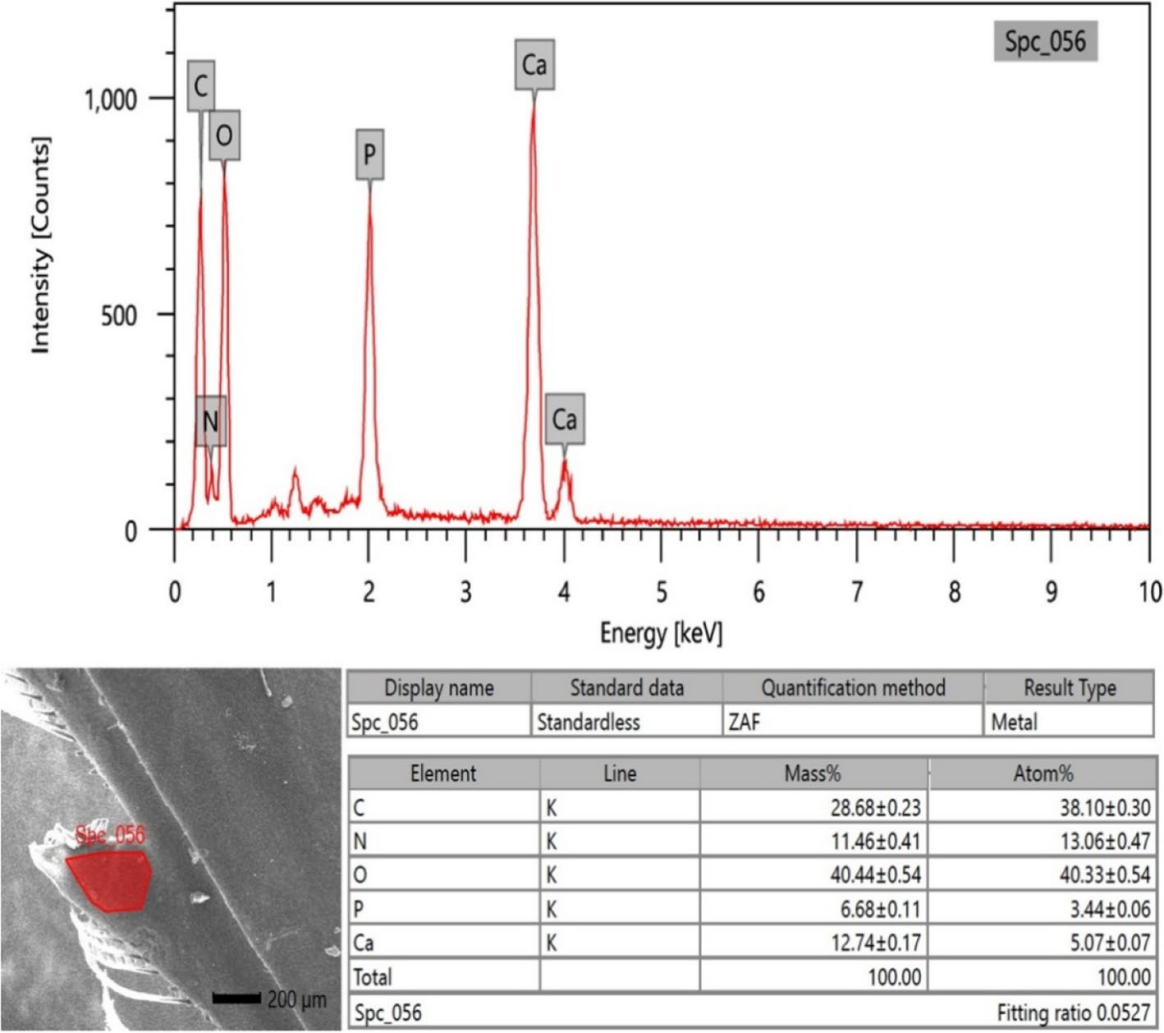
SEM–EDX image depicting the elemental composition of the external surface of the rostrum cuticle in *Marsupenaeus japonicus*

**Fig. 12:**
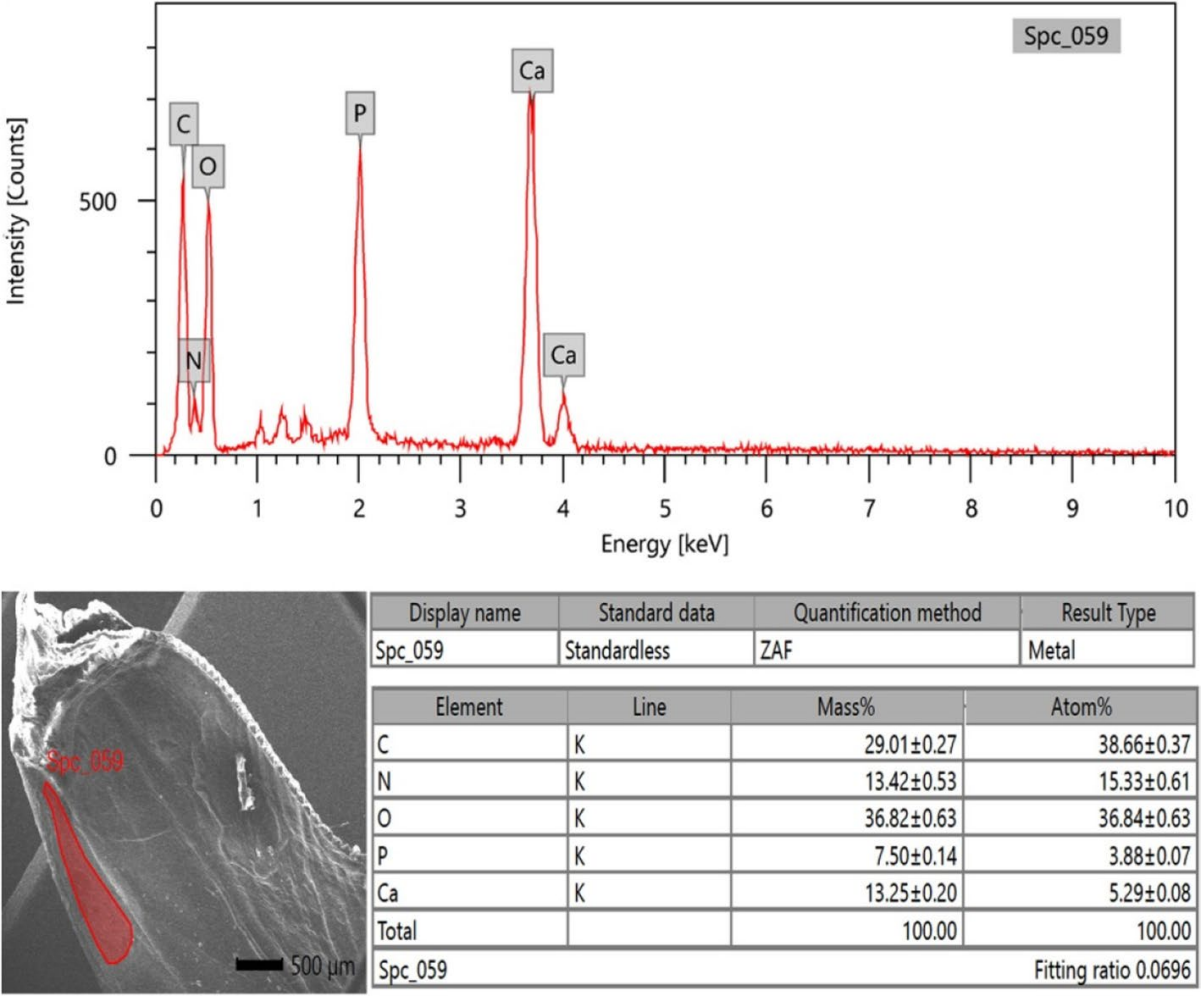
SEM–EDX image showing the elemental composition of the external surface of the spear-like cuticle of the scaphocerite in *Marsupenaeus japonicus*

Micro-elemental analysis of the cuticle's external surface was conducted on soft structures such as the antenna and hard structures like the rostrum and the spear-like shape of the scaphocerite. The analysis involved collecting photon energy dispersive X-ray spectra from three different parts of the external surface of the cuticle, conducted at an accelerating voltage of 20 keV (Figs. 10, 11, and 12). The EDX spectra documented the elements by mass percentage (%) and identified multiple elements in three different parts (Table 2). At the antenna (soft structure), the most prominent elements, ranked in descending order of concentration, were oxygen, carbon, nitrogen, calcium, phosphorus, and very trace concentrations represented by magnesium, sodium, and aluminum. For the rostrum, the most prominent elements, ranked in descending order of concentration, were oxygen, carbon, calcium, nitrogen, and phosphorus, where the calcium percentage was higher than the nitrogen percentage. In the spear-like shape of the scaphocerite, the most prominent elements, ranked in descending order of concentration, were oxygen, carbon, nitrogen, calcium, and phosphorus. The higher percentages of calcium and phosphorus were presented at the spear-like shape of the scaphocerite, rostrum, and antenna (20.75%, 19.42%, and 15.99%), respectively. Additionally, the higher percentage of oxygen was located at the rostrum, the higher percentage of carbon was located at the antenna, and the higher percentage of nitrogen was located at the spear-like shape of the scaphocerite.

**Table 2:** Mass percentage, mean, and standard error of different elements on the external surface of the cuticle in the antenna, rostrum, and spear-like shape of the scaphocerite in *Marsupenaeus japonicus*

	**Antenna**	**Rostrum**	**Spear-like shape of Scaphocerite**
C	30.43 ± 0.22	28.68 ± 0.23	29.01 ± 0.27
N	12.82 ± 0.39	11.46 ± 0.41	13.42 ± 0.53
O	39.38 ± 0.48	40.44 ± 0.54	36.82 ± 0.63
P	6.66 ± 0.10	6.68 ± 0.11	7.50 ± 0.14
Ca	9.33 ± 0.13	12.74 ± 0.17	13.25 ± 0.20
Na	0.41 ± 0.04		
Mg	0.73 ± 0.04		
Al	0.24 ± 0.03		

## Discussion

### Morphological analysis of *Marsupenaeus* japonicus shrimp

We examined *Marsupenaeus japonicus* shrimp with a length range of 11.7 to 13.8 cm, representing the adult stage available for fishing in Damietta Governorate. Our specimens were consistent with findings indicating that adult, healthy *Marsupenaeus japonicus* shrimp typically weigh around 15 g and measure between 10 and 12 cm in length, obtained from a seafood market in Hangzhou [16]. The *Marsupenaeus japonicus* shrimp is omnivorous with a significant carnivorous component, feeding primarily on crustacean and fish remnants [61].

### Comparative morphological characteristics of antenna and antennule structure in *Marsupenaeus* japonicus and other shrimp: Segments, flagella, and setae

*Marsupenaeus japonicus* antennae had four basal segments and a fragile single flagellum, like exotic lysmatid shrimp [62], while L. vannamei's second antenna had three basal segments and a single flagellum longer than the body [34]. In the morphological study of five shrimp species, namely *Palaemon elegans, Mirocaris fortunata, Rimicaris exoculata, Chorocaris chacei, and Alvinocaris markensis*, both antennae and antennules exhibited a similar structure comprising a peduncle and segmented flagella. The antennae had one flagellum, while the antennules possessed two – an outer or lateral flagellum and an inner or medial flagellum. Across the three flagella, the annuli displayed variations in diameter and length. Specifically, the annuli were large and short at the base, gradually becoming thinner and longer toward the apex. These morphological characteristics were observed in the study conducted by [63]. In our study, the structure of the antennules peduncles in *Marsupenaeus japonicus* differed from the general pattern observed in the comparison with [63]. Specifically, the antennules peduncles in our specimens had three segments, predominantly covered by plumose setae and terminating in a thin and long medial flagellum alongside a thick lateral flagellum. Aesthetascs were present on both sides of the medial flagellum, while the lateral flagellum exhibited a covering of plumose and simple setae. Contrary to the findings of [63], we observed pappose setae on the rostrum and eye stalk of *Marsupenaeus japonicus*. These pappose setae were suggested to have a mechanical function, potentially contributing to grooming activities. Grooming behaviors in crustaceans are often associated with various setal types, including simple, serrate, pappose, and scaled setae, highlighting the diverse roles these structures may play in maintaining the cleanliness and integrity of different body regions [64].

The antennule is uniquely adapted to function throughout the life of the shrimp in response to an ever-changing environment. It contains specialized secretory glands, grooming mechanisms, and behaviors designed to keep it functional and clean. The antennular sensilla and their associated annuli are periodically replaced and renewed with each molt, resulting in the complete regeneration of the antennule. As a result, the antennule plays a vital role as a dynamic and complex sensory-motor organ, actively participating in various essential aspects of crustacean life [65].

### Comparative insights on the fine structure of antennal and antennular setae in *Marsupenaeus* japonicus and other decapod crustaceans

SEM is a helpful tool for describing the fine structure of the cephalic part of the *Marsupenaeus japonicus*. The use of SEM on the cuticle outgrowths called setae in crustaceans has proven beneficial for understanding both their sensory functions and taxonomic classifications. Specifically, these plumose setae assist crustaceans in detecting water currents [36].

When comparing *Marsupenaeus japonicus* to other decapod crustaceans, it is noteworthy that there are variations in the number and type of setae found on the antennules and antennal setae [66]. Aesthetascs, which are antennule-specific setae, are largely assumed to function as chemoreceptors [67]. The number and length of these setae vary among crustacean species, demonstrating important morphological adaptations [36, 68]. Setae are distributed throughout the body and provide an overview of some of the prominent sensors on the chemosensory organs that contribute to feeding behavior [25, 67, 69].

Chemoreceptive setae and their various functions are essential for detecting potential food sources and mates [67, 69, 70]. For instance, the lateral flagellum of the antennules contains unimodal, olfactory aesthetasc used in distance chemoreception [31]. On the other hand, the long flagella of the second antennae are thought to be chemotactic, equipped with bimodal, gustatory receptors [25]. The gustatory chemosensory system is partially dependent on the bimodal antennal setae receptors, which interact with relevant molecules and generate receptor and action potentials, transmitting the effects of chemical stimuli [70, 71].

Moreover, it is observed that many species' antennae exhibit sexual dimorphism, presumably as an adaptation for efficient mate searching. For example, the chemical signals involved in the mate recognition system of *Palaemonetes pugio* shrimp highlight the specialized receptors males use to receive and interpret female signals [24].

In *Marsupenaeus japonicus*, five distinct types of antennal and antennular setae are identified, compared to one aesthetasc and three other types found in *Lysmata* [72]. Similarly, *Alpheus heterochaelis* (snapping shrimp) exhibits six setae [73], while the Caribbean spiny lobster, *Panulirus argus*, demonstrates a total of ten setae [74]. As discovered in various species of crayfish [75], prawns [76], and Lysmata shrimp [34, 72], the structure and the number of antennal or antennular setae can vary among correlated species, influenced by different physical environments, such as the type of water and social environments.

Although antennules may differ in size, their functions remain similar. For instance, the chemosensory control of feeding in *Litopenaeus vannamei*, a crustacean with small antennules that thrives in both pelagic and benthic environments, follows a sequence similar to that observed in larger-antennuled, benthic species such as spiny lobsters (Achelata), crayfish (Astacidea), and crabs (Meirua). Despite the differences in antennule size and habitat, these species exhibit analogous feeding behaviors, with their antennules playing a vital role in chemosensory mechanisms that allow them to detect food and other chemical cues in their environment [34].

### Plumose setae presence in *Marsupenaeus* japonicus and its functional implications

We observed the presence of plumose setae in the antennules, rostrum, eye stalks, and scaphocerite of *Marsupenaeus japonicus*. [77] highlighted that plumose setae contribute to an increase in the surface area of sensory appendages, emphasizing their significance in understanding the sensory capabilities of crustaceans. Specifically, plumose setae located on natatory appendages, such as pleopods, are specialized structures crucial for sensory perception. Importantly, water-soluble dyes are not able to penetrate plumose setae, indicating their role in sensory function. Our findings suggest that the external morphology of setae, including plumose setae, serves as a valuable indicator of their specific functional roles, such as feeding, grooming, and locomotion. Understanding the presence and distribution of plumose setae across different structures provides insights into the multifaceted sensory functions exhibited by crustaceans.

### Dimensional variation of setae across decapod species

Our study thoroughly examined setae in *Marsupenaeus japonicus* and revealed notable variations in length and width within the same seta type across different organs. The diverse dimensions observed across examined structures highlight the intricacies of setal morphology. Specifically, in male *Marsupenaeus japonicus*, the antennules exhibited three types of setae, namely Aesthetasc, simple, and plumose, while the antenna featured other types, including unipennate, filamentous, and simple setae. Our findings contrast those observed in decapod shrimps, where males lack unique setal types on the second antennae (antennal) flagella. In decapod shrimps, the antennal flagella are chemotactic, and their setae are proposed as sensilla involved in the recognition of females by males through contact with a sex pheromone on the female's surface [78]. However, our results align with those reported in the giant river prawn *Macrobrachium rosenbergii*, where antennules and antennae possess three types of setae. Two setae types in *Marsupenaeus japonicus*, namely aesthetascs and simple plumose, are similar to those in *Macrobrachium rosenbergii.* The third type in *Marsupenaeus japonicus* is plumose, while *Macrobrachium rosenbergii* has pappose setae [79].

Notably, our study revealed that aesthetasc setae in *Marsupenaeus japonicus* had a length of 390.8 ± 20.2 µm and a width at the base of around 20.8 ± 1.02 µm. These dimensions differ from those reported in other studies, such as [63], which indicated variations in aesthetasc setae dimensions along the flagella, being thinner and shorter at the base and growing toward the apex. Furthermore, [79] highlighted that setae increased in density, covered surface, and distribution during ontogeny, suggesting a relationship between chemoreception and mechanoreception. Our observations contribute to understanding setal diversity in *Marsupenaeus japonicus* and provide insights into potential functional roles related to sensory mechanisms.

In our study on *Marsupenaeus japonicus*, the dimensions of aesthetasc setae (diameter × length) were found to be 20.8 × 390.8 μm. Comparatively, *in Lysmata b*, the dimensions were 20 × 800 μm [80]. Other decapod species showed varying dimensions, such as *Palaemon elegans* with 14 × 230 μm, *Mirocaris fortunata* with 16 × 234 μm, *Rimicaris exoculata* with 20 × 170 μm, *Chorocaris chacei* with 19 × 251 μm, and Alvinocaris markensis with 21 × 531 μm [63]. In crabs, *Callinectes sapidus* exhibited aesthetasc setae dimensions of 12 × 795 μm, while lobsters like *Panulirus argus* had larger dimensions of 40 × 1000 μm [81, 82]. These variations in aesthetasc setae dimensions across different decapod species underscore the diversity in sensory structures among crustaceans.

### Adaptations of shrimp for defense: An examination of key structures

The defense mechanisms of shrimp involve the utilization of various structures. The rostrum and chelae are crucial components in defense strategies [20, 21]. Additionally, anterior and posterior carapace spines have been shown to play a role in deterring predators [22]. The firmly extended spinous scaphocerite is proposed as a defense mechanism by increasing the required gape of the predator's mouth [23]. Furthermore, the telson has been recognized as a crucial defense tool in shrimp [83]. Our study aims to contribute to understanding specific head structures, such as antennules, antenna, scaphocerite, rostrum, and eye stalk, in the context of shrimp's defense mechanisms.

### Defense tools in *Marsupenaeus* japonicus with comparative insights from other species

In our work, we propose several macro- and micro-structures that serve as defense tools. The first tool is a moderate-length, spear-shaped rostrum located at the middle and dorsal side of the cephalic region. This rostrum has one ventral spine and 9–10 dorsal serrations angled rostro-dorsally. The rostrum also includes different types of setae **(**simple setae, plumose setae, and pappose setae**)**, which function as sensory tools, acting like radar to detect threats. Similarly, the long and short rostrums of *Xiphocaris elongata* provide effective antipredator defenses against *Agonostomus monticola* during different phases of predator–prey interactions, underscoring the rostrum’s key role in defense mechanisms [21]. Additionally, the plumose setae play a critical role in detecting water currents, further improving the shrimp’s ability to sense environmental changes and respond to potential threats [36].

The second tool consists of spiny structures that project from various parts of the shrimp. These include the short orbital spine, long wedge-shaped antennule spine extending from the carapace behind the eye stalk and antenna, tooth structures at the basicerite 1st segment of the antenna, and an additional lateral projection stylocerite at the 1st segment of antennules. These spiny structures significantly enhance the shrimp’s ability to deter predators through mechanical defense.

The third tool is the scaphocerite, which has a spear-like shape with a tapered end. Its firmly extended spinous structure acts as a defense mechanism, as observed in *Oplophorus* species [23]. Collectively, these structural adaptations highlight the shrimp’s evolutionary responses to ecological pressures, enhancing its survival in predator-rich environments.

### Comparative analysis of the defensive morphological adaptations in the rostrum of *Marsupenaeus* japonicus and other shrimp species

The rostrum of *Marsupenaeus japonicus* from Bardawil Lagoon had 9–10 teeth on the dorsal side and one tooth on the ventral side [1]. The rostrum is covered with 8–10 spines on top and one or two on the bottom [13, 18]. The dorsal part had 9–11 teeth, while the ventral part had one tooth [84]. In the *Marsupenaeus japonicus* we studied, there were various types of setae at the eye stalk and pores. The sensory tubercle on the eyestalk of the shrimp *Crangon crangon* had sensory pores and eye tubercle sensory organs with complex structures and at least two chemoreceptor-like organs at the pores [85]. Moreover, the ultrastructure of the sensory pore at the eye stalk in Crustacea natantia explained the presence of a layer of supporting cells enveloped by the terminal parts of sensory cell bodies, indicating a sophisticated sensory system [86].

In our study, the rostrum of *Marsupenaeus japonicus* displayed distinct characteristics, with 9–10 dorsal spines and a single ventral spine before its pointed end, aligned with the middle of the second segment of the antennule peduncle. Lateral grooves and ridges flank the dorsal spines, which are obliquely directed rostrally, with an angle ranging from 35 to 45 degrees. This structural configuration of the rostrum is hypothesized to serve a defensive function, possibly enhancing the shrimp’s ability to deter predators or engage in territorial disputes. The oblique arrangement of the spines may further contribute to effective defense strategies, emphasizing the diverse evolutionary adaptations of crustaceans in response to ecological pressures.

When comparing our findings to other species, distinct variations in rostral morphology become evident. For instance, in *Macrobrachium mammillodactylus*, the rostrum features evenly spaced teeth, with 8–14 dorsal teeth and 3–6 ventral teeth [87, 88]. Similarly, in *Penaeus semisulcatus*, the rostrum contains 5–8 teeth on the dorsal side and 2–4 teeth on the ventral side [1]. These morphological differences across species suggest that rostrum adaptations are tailored to specific functional roles, further highlighting the ecological and evolutionary diversity within the Crustacea.

### Structural variations and functional roles of the scaphocerite in shrimp defense mechanisms

The scaphocerite, a pivotal crustacean appendage, exhibits notable structural variations among species. In our examination of *Marsupenaeus japonicus*, we uncovered a distinctive composition featuring two distinct sections: a long oval with a lateral edge and a spear-like shape with a tapered end. The rostral part exhibited a semicircular form with oblique folds on the dorsal surface near its medial side, emphasizing its intricate nature. Medial margins were adorned with long plumose setae and a pocket extension articulated with these setae, suggesting a multifaceted role, potentially acting as paddles. Additionally, wavy folds on the dorsal surface contribute to swimming balance. Our findings align with studies indicating that the scaphocerite, telson, and uropods serve as swimming appendages [89], consistent with their structural adaptations for this purpose. Unlike the whiteleg shrimp, where the scaphocerite primarily functions as a lateral stabilizing fin during escape reactions [90], our results suggest that in *Marsupenaeus japonicus*, the scaphocerite's shorter length implies a less critical role in balance or orientation.

#### Elemental composition and functional analysis of cephalothoracic structures in *Marsupenaeus* japonicus: Insights from EDX analysis

In our study, we utilized EDX analysis to unveil elemental composition variations within specific structural components of *Marsupenaeus japonicus*, providing valuable insights into the intricacies of its exoskeleton. Our focused examination centered on the spear-like shape of the scaphocerite, rostrum, and antenna. While prior studies on crustaceans, such as those by [91, 92], unveiled general composition findings, our approach offered a more detailed perspective on the elemental makeup of key head structures. In contrast to the findings of [91], which emphasized calcite and trace elements like nitrogen and silicon on shrimp shell surfaces, our results indicated elevated percentages of calcium and phosphorus in the spear-like structures of scaphocerite, rostrum, and antenna (20.75%, 19.42%, and 15.99%, respectively). These observations suggest heightened hardness in these specific regions, hinting at their potential role in defense mechanisms. This aligns with the recognized anatomical features of the rostrum, underscoring its critical role in defense and mechanical support.

Our findings diverged in comparison with [92], who highlighted the chitin biopolymers and CaCO3 composition in crustacean exoskeletons. Our quantitative EDX data underscored the prevalence of carbon, oxygen, and calcium in the examined shrimp exoskeleton. The increased calcium phosphate in the relatively thick impact region implies enhanced hardness and stiffness, contributing to efficient energy transfer during interactions with prey or conspecifics. This supports the notion that specific exoskeletal regions in *Marsupenaeus japonicus* are adapted for defensive purposes. Examining the elemental composition of other crustaceans, such as *Rimicaris exoculata* and *Pandalus platyceros* [93, 94], they found distinct calcium and phosphorus atomic percentages in their exoskeleton cross sections, aligning with our results. Our findings, indicating a higher concentration of carbon at the antenna of *Marsupenaeus japonicus*, suggest a substantial presence of carbon-based compounds, potentially contributing to the flexibility or functionality of this structure. Our focused investigation on *Marsupenaeus japonicus* contributes valuable insights into the elemental composition variations within specific head structures, enriching the broader understanding of adaptive and defensive features in crustaceans. While our current research focused primarily on the surface morphology and elemental composition of the cephalothoracic structures, our future plan is to complement these findings with histomorphological and light microscopy analyses to gain deeper insights into the cephalothoracic structures of *Marsupenaeus japonicus*.

## Conclusion

Our study represents the first comprehensive analysis of the cephalothoracic structures of *Marsupenaeus japonicus* combining SEM and EDX techniques, marking a novel contribution to crustacean biology. By correlating detailed morphological features with elemental composition, our research offers new insights into the functional roles of these structures, particularly in chemoreception and defense mechanisms. The investigation focused on the gross morphology and morphometric features of key structures, such as antennules, antennae, scaphocerite, rostrums, and eye stalks, shedding light on their functions, particularly in chemoreception and defense. The thorough examination of setae across these structures highlighted their diverse lengths and widths, indicating specialized functions. The antennules and antennae, adorned with plumose setae and distinctive flagella, were shown to be crucial for sensory reception. The scaphocerite, resembling a paddle, and the rostrum, with its dorsal spines and unique seta arrangements, were identified as potentially significant in defensive mechanisms. Elemental composition analysis through EDX unveiled higher percentages of calcium and phosphorus in the spear-like structures of the scaphocerite, rostrum, and antenna (20.75%, 19.42%, and 15.99%, respectively). This suggests elevated hardness in these regions, supporting their role in defense. The compound eyes, characterized by a honeycomb cornea connected to an optic stalk adorned with plumose setae, further contribute to the understanding of the sensory capabilities of Kuruma shrimp. Overall, these findings provide valuable insights into the complex morphological features and functional roles of the cephalothoracic region in *Marsupenaeus japonicus.* This study not only enhances our understanding of the species but also opens new avenues for future research by demonstrating the value of integrating SEM and EDX techniques for studying crustacean morphology, with promising applications in aquaculture and marine ecology.

## Data Availability

The datasets used and analyzed during the current study are available from the corresponding author upon reasonable request.
